# A Transcriptomic Analysis of Bottle Gourd-Type Rootstock Roots Identifies Novel Transcription Factors Responsive to Low Root Zone Temperature Stress

**DOI:** 10.3390/ijms25158288

**Published:** 2024-07-29

**Authors:** Jinqiu Liu, Man Zhang, Jian Xu, Xiefeng Yao, Lina Lou, Qian Hou, Lingli Zhu, Xingping Yang, Guang Liu, Jinhua Xu

**Affiliations:** 1 Institute of Vegetable Crop, Jiangsu Academy of Agricultural Sciences, Nanjing 210014, China; 20210092@jaas.ac.cn (J.L.); 20110041@jaas.ac.cn (M.Z.); xjian.1990@163.com (J.X.); 20100039@jaas.ac.cn (X.Y.); 20120053@jaas.ac.cn (L.L.); 20090987@jaas.ac.cn (Q.H.); 20080992@jaas.ac.cn (L.Z.); 19830012@jaas.ac.cn (X.Y.); 2 Laboratory for Genetic Improvement of High Efficiency Horticultural Crops in Jiangsu Province, Nanjing 210014, China

**Keywords:** bottle gourd, root, low root zone temperature, RNA-Seq, transcription factor

## Abstract

The bottle gourd [*Lagenaria siceraria* (Molina) Standl.] is often utilized as a rootstock for watermelon grafting. This practice effectively mitigates the challenges associated with continuous cropping obstacles in watermelon cultivation. The lower ground temperature has a direct impact on the rootstocks’ root development and nutrient absorption, ultimately leading to slower growth and even the onset of yellowing. However, the mechanisms underlying the bottle gourd’s regulation of root growth in response to low root zone temperature (LRT) remain elusive. Understanding the dynamic response of bottle gourd roots to LRT stress is crucial for advancing research regarding its tolerance to low temperatures. In this study, we compared the physiological traits of bottle gourd roots under control and LRT treatments; root sample transcriptomic profiles were monitored after 0 h, 48 h and 72 h of LRT treatment. LRT stress increased the malondialdehyde (MDA) content, relative electrolyte permeability and reactive oxygen species (ROS) levels, especially H_2_O_2_ and O^2−^. Concurrently, LRT treatment enhanced the activities of antioxidant enzymes like superoxide dismutase (SOD) and peroxidase (POD). RNA-Seq analysis revealed the presence of 2507 and 1326 differentially expressed genes (DEGs) after 48 h and 72 h of LRT treatment, respectively. Notably, 174 and 271 transcription factors (TFs) were identified as DEGs compared to the 0 h control. We utilized quantitative real-time polymerase chain reaction (qRT-PCR) to confirm the expression patterns of DEGs belonging to the WRKY, NAC, bHLH, AP2/ERF and MYB families. Collectively, our study provides a robust foundation for the functional characterization of LRT-responsive TFs in bottle gourd roots. Furthermore, these insights may contribute to the enhancement in cold tolerance in bottle gourd-type rootstocks, thereby advancing molecular breeding efforts.

## 1. Introduction

Low-temperature stress is one of the most debilitating abiotic stresses in agriculture, as it significantly affects plant growth, development, quality, yield and geographical distribution. Furthermore, it can inflict irreversible damage on both plant yield and quality [1,2]. Low temperatures induce alterations in plant cell membrane structure and stability and lead to chloroplast photo-oxidative damage by increasing the production of reactive oxygen species (ROS) such as hydroxyl radicals (OH), superoxide anion radical (O^2−^) and hydrogen peroxide (H_2_O_2_) [3,4]. Low-temperature stress can be categorized into chilling stress (0 °C–15 °C) and freezing stress (<0 °C), which can be further divided into low air temperature and low root zone temperature (LRT) [5]. In agricultural production, LRT stress frequently occurs in the seedling stage and early stages of planting after the sowing of crops in winter and early spring, which seriously affect the growth and development of crop roots [6,7]. LRT can directly alter plant root system architecture (RSA), which has been defined as the geometric description of the shape of a root system [8]. LRT traits including the length, density and spatial structure of the primary root, lateral root and root hair, etc., affect nutrient transportation, water absorption and a plant’s adaptability to the environment. LRT subsequently affects root functions, ranging from the acquisition of nutrients to hydraulic conductance, which results in slow growth and a decline in the yield, quality and development of a crop [9,10,11].

Recently, the persistent challenges posed by continuous cropping have emerged as a significant hindrance to the progress and development of the watermelon industry [12]. Grafting is currently the most effective technical measure for reducing the occurrence of continuous cropping obstacles in production [13,14]. Due to its strong root system architecture and strong affinity for grafting with watermelon, the bottle gourd is commonly used as a grafting rootstock for watermelon and other melon crops to defend against soil-borne fungal pathogenic diseases (e.g., *Fusarium* wilt) and to increase abiotic stress tolerance (e.g., salt and drought) [15,16,17]. The bottle gourd [*Lagenaria siceraria* (Molina) Standl.], with a chromosomal count of 2*n* = 2*x* = 22, belongs to the genus *Lagenaria* within the *Cucurbitaceae* family [18,19]. However, the bottle gourd originated in sub-Saharan Africa, and it is a thermophilic crop that is not tolerant of low temperature [20]. Strong roots are the primary condition for the breeding of grafted rootstocks of melon crops. At the same time, the strong roots of the bottle gourd should also have the ability to tolerate low-temperature stress. However, little is known about the root traits of the bottle gourd under low-temperature stress. Therefore, enhancement in cold tolerance remains one of the major breeding goals in bottle gourd type rootstock.

Dicotyledonous roots (e.g., in *Arabidopsis thaliana*) are composed of a primary root and lateral roots, while monocotyledonous roots (e.g., in rice and maize) are adventitious roots that include embryonic taproots and embryonic seed roots forming lateral roots [4,21]. The discovery and regulation mode of genes related to root growth and development under medium and low-temperature stress have been studied in depth. At present, only pumpkin-type rootstocks used for watermelon grafting have been reported in studies on their root growth and development under low-temperature stress [22]. Many factors have been shown to impact low-temperature tolerance in the roots of crops: transcription factors (TFs) [23,24], phytohormones [25,26], ROS [3], microRNA [27,28], long non-coding RNA (lncRNA) [29] and metabolic genes [30,31]. TFs perform their biological functions by regulating the transcription of target genes and play key roles in regulating plant root growth, development and stress response [32,33]. Plant TFs can regulate low-temperature resistance by simultaneously controlling multiple genes via transcriptional cascades in the cold response pathway, such as genes related to ROS signaling and phytohormone signaling (e.g., abscisic acid, auxin, jasmonic acid) [1,34]. Thus, TFs can be promising targets for the breeding of low-temperature crops or cold-tolerant roots.

Plant TFs regulate root architecture by regulating multiple genes or plant hormone levels in the low-temperature response pathway, thereby giving plants the ability to tolerate low-temperature stress. There are two modes of plant response to low-temperature stress. One is the C-repeat binding factor (CBF)-dependent TF regulation mode, that is, the ICE (INDUCER OF CBF EXPRESSION 1)-CBF-COR (COLD REGULATED) mode that regulates plants’ cold tolerance [35,36]; the other is some members of other transcription factors (such as NAC (NAM/ATAF1/2/CUC2), WRKY (WRKYGOK), basic leucine zipper (bZIP), MYB (myb avian myeloblastosis viral oncogene homolog) and AP2/ERF (APETALA2/ethylene responsive factor), etc.), which participate in the regulation of plants’ low-temperature responses through a CBF-independent pathway. Rice OsNAC45 regulates peroxidase activity and lignin biosynthesis in roots under cold conditions and, at the same time, alleviates cold-induced root growth inhibition by reducing the ABA content in roots [37]. GmNAC20, a soybean protein, serves as a positive regulator in enhancing cold resistance and promoting lateral root development in transgenic rice plants [23,38]. Similarly, among WRKY types, TFs have been identified as important players in cold tolerance and low-temperature responses. In bermudagrass, an overexpression of *CdWRKY2* enhanced the cold tolerance and hairy root numbers of transgenic *Arabidopsis* and bermudagrass [39]. This confirmed that TFs play an important role in regulating root development in response to low-temperature responses in plants. However, responses of shoot and root physiology, transcription, and metabolism to LRT have rarely been studied. Additionally, few TF families have been functionally characterized as regulators controlling low-temperature responses in bottle gourd roots. Here, we performed a genome-wide RNA sequencing of bottle gourd roots under 15 °C LRT stress. We laid the foundation for the further analysis of differentially expressed gene (DEG) TFs and the mechanism by which LRT regulates the lateral root growth of the bottle gourd. Subsequently, this will provide a theoretical basis for preventing LRT from affecting bottle gourd roots.

## 2. Results

### 2.1. LRT Stress Has a Major Impact on the Bottle Gourd Root

**Figure 1:**
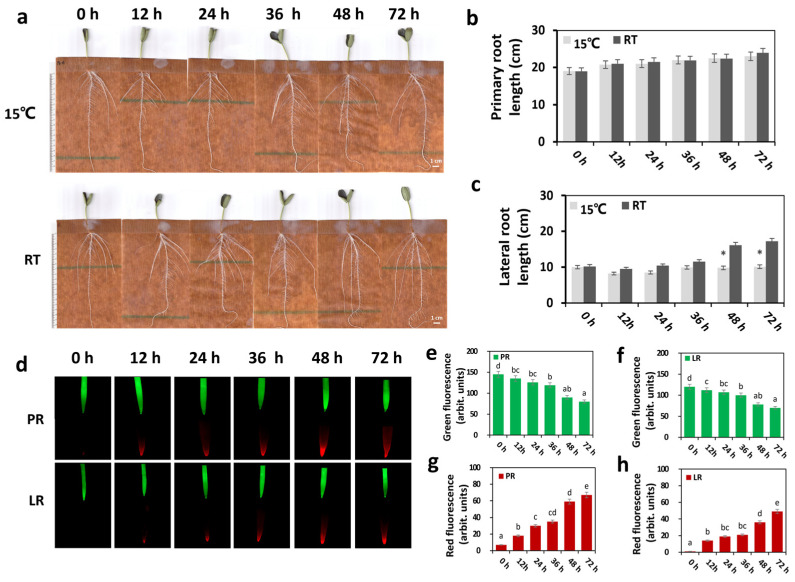
Detached Chaofeng F1 root in response to LRT treatment. (**a**) Phenotypes of the Chaofeng F1 root under LRT stress; (**b**,**c**) Lengths of the root ((**b**), primary root; (**c**), lateral root) were treated after LRT (0 h, 12 h, 24 h, 36 h, 48 h and 72 h) and control treatments (* *p* < 0.05). (**d**) Cell viability staining of the PR and LR apex zone of Chaofeng F1. Plants were treated with LRT treatment (0 h, 12 h, 24 h, 36 h, 48 h and 72 h). (**e**,**f**) Quantified intensity of the green ((**e**), PR; (**f**), LR) fluorescent signal. (**g**,**h**) Quantified intensity of the red ((**g**), PR; (**h**), LR) fluorescent signal. Values are expressed as the mean ± SE (*n* = 6); letters indicate a significant difference between means (*p* < 0.05) according to Duncan’s multiple range tests.

Chaofeng F1 is variety of bottle gourd that exhibits a strong tolerance to low temperature. In order to investigate the LRT response in bottle gourd roots, Chaofeng F1 roots’ phenotypical characteristics were investigated in pouches at the post-germination stage under 15 °C LRT stress for a period of 72 h. In contrast to the control, the lateral root length (LRL) decreased significantly from 12 h, but the primary root length (PRL) did not change significantly (Figure 1a–c). To track the physiological changes caused by LRT stress in bottle gourd roots, the root activity (primary root, PR, and lateral roots, LRs) was measured from 0 h to 72 h during 15 °C LRT treatment. As shown in Figure 1d, the root activity of both the PR and LRs of the bottle gourd significantly decreased from 12 h to 72 h under 15 °C LRT stress, in contrast to at 0 h. Specifically, after 48 h, a noticeable decline in the number of living cells and a corresponding increase in the number of dead cells were observed in the roots (Figure 1e–h), aligning with the phenotype changes observed in Figure 1a. Above all, these findings indicate that 15 °C LRT stress strongly impacts the bottle gourd root’s phenotypical and root activity.

### 2.2. LRT Affects Bottle Gourd Roots’ Physiological Traits

**Figure 2:**
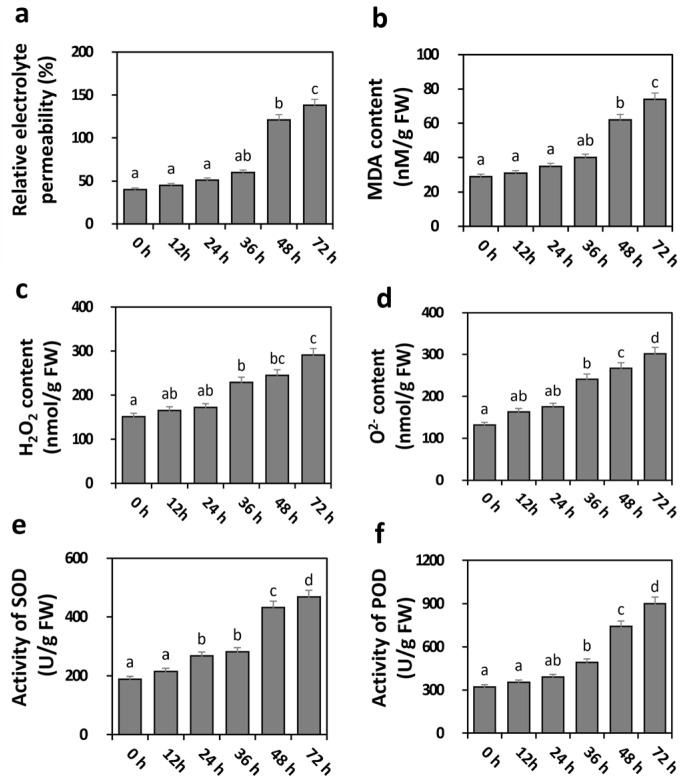
Physiological traits in response to LRT treatment in Chaofeng F1. The (**a**) relative electrolyte permeability, (**b**) MDA content, (**c**) H_2_O_2_ content, (**d**) O^2−^ content, (**e**) SOD activity and (**f**) peroxidase activity were determined in the detached root of Chaofeng F1 after 0 h, 12 h, 24 h, 36 h, 48 h and 72 h LRT treatment. Values are expressed as the mean ± SE (*n* = 6); letters indicate a significant difference between means (*p* < 0.05) according to Duncan’s multiple range tests.

To track the physiological changes caused by LRT stress in bottle gourd roots, the relative electrolyte permeability and MDA content were measured from 0 h to 72 h in the 15 °C LRT treatment. As shown in Figure 1a, the relative electrolyte permeability of the bottle gourd root increased gradually over the duration of the treatment and was significantly higher after 48 h of LRT in contrast to that at 0 h. In addition, the content of MDA quantified after 36 h, 48 h and 72 h of LRT was significantly higher than that at 0 h (Figure 2b). It has been shown that reactive oxygen species (ROS) and oxidative stress can be triggered by the shaping of plant root architecture under LRT [3,40]. To verify whether LRT treatment induced oxidative stress and ROS stress in bottle gourd roots, we analyzed the content of H_2_O_2_ and O^2−^; a gradual increase in both was observed at the earlier time points, and a significant increase was observed after 36 h of LRT (Figure 2c,d). The changes in the activities of superoxide dismutase (SOD) and peroxidase (POD) were significantly enhanced after 48 h of LRT (Figure 2e,f). In addition, with the increase in time, the content of H_2_O_2_, O^2−^, POD and SOD increased gradually. Taken together, these results indicate that 15 °C LRT stress triggered oxidative stress and the roots’ physiological traits.

### 2.3. Transcriptomic Analysis Reveals DEGs in Response to LRT Stress

**Figure 3:**
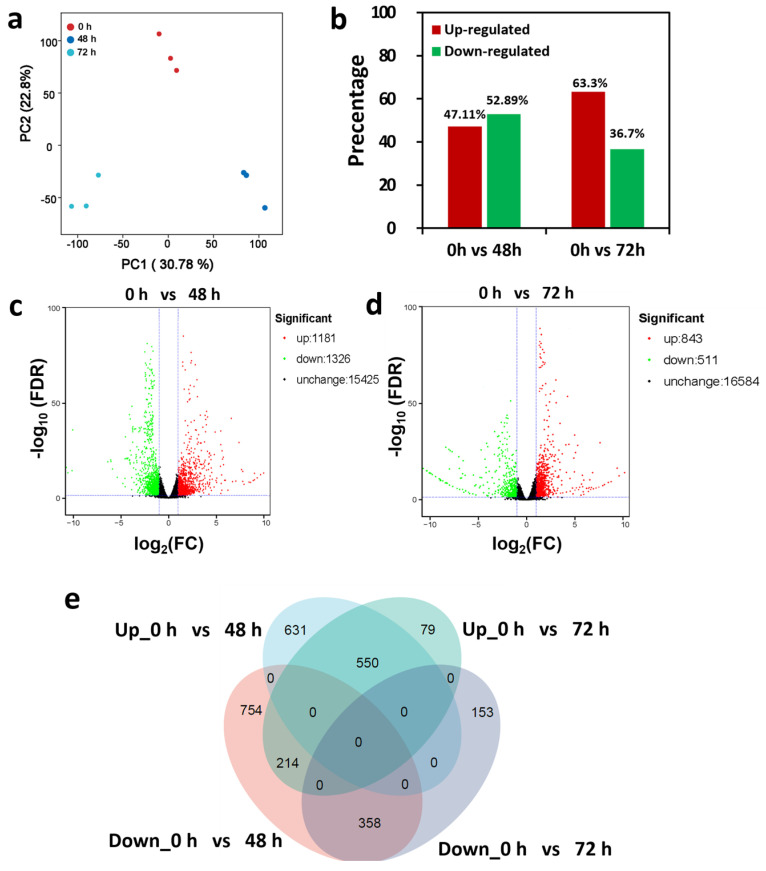
Transcriptomic profiling analysis of roots in response to LRT stress. (**a**) Principal component analysis (PCA) of RNA-Seq data of the Chaofeng F1 root in response to LRT stress. (**b**) Percentages of upregulated and downregulated DEGs among total DEGs detected in 48 h and 72 h LRT-treated vs. 0 h roots. (**c**,**d**) Volcano plots of LRT-responsive DEGs in the Chaofeng F1 root after (**c**) 48 h and (**d**) 72 h LRT-treated vs. 0 h roots. (**e**) Venn diagrams of up- and downregulated DEGs at 0 h, 48 h and 72 h across four LRT treatment time points.

To explore the DEGs specifically responding to LRT tolerance of the bottle gourd roots, we profiled the root transcriptome of Chaofeng F1 treated with 15 °C LRT for 0 h, 48 h and 72 h by RNA-Seq. As shown in Figure 3a, the principal component analysis (PCA) showed that the LRT treatment groups of 48 h and 72 h were clearly separated from the 0 h group, and the PC1 and PC2 values were 30.78% and 22.80%, respectively. This suggests a good correlation among the biological relocates. In response to LRT stress, RNA-Seq analysis detected differentially expressed genes (DEGs), with 47.11% and 63.30% of all DEGs identified after 48 h and 72 h of LRT, respectively, in contrast to 0 h which had upregulated genes, and the rest, of which 52.89% and 37.3% were downregulated genes (Figure 3b). Overall, 2507 (1181 upregulated and 1326 downregulated) and 1354 (843 upregulated and 511 downregulated) DEGs were found at 48 h (Figure 3c) and at 72 h (Figure 3d) compared with the 0 h control, respectively; in addition, 15,425 and 16,584 genes were not differentially expressed in this comparison.

The Venn diagram (Figure 3e) shows that 358 genes were commonly downregulated and 550 commonly upregulated in two comparisons: 48 h vs. 0 h and 72 h vs. 0 h. Furthermore, 214 genes were downregulated after 48 h of LRT but upregulated after 72 h of LRT. Similarly, in contrast to the 0 h control, we also identified numerous unique DEGs, including 746 genes that were downregulated only after 48 h of LRT, 153 genes that were downregulated only after 72 h of LRT, 631 genes that were upregulated only after 48 h of LRT, and 79 genes that were only upregulated after 72 h of LRT stress.

Taken together, the above results suggest that the bottle gourd roots’ response to LRT stress at the transcriptional level is a dynamic process and that it has evolved into a series of common genes and DEGs at different time points at a transcriptomic level.

### 2.4. Gene Ontology (GO) and Kyoto Encyclopedia of Genes and Genomes (KEGG) Enrichment Analysis among DEGs Involved in Translation and the Stress Response

**Figure 4:**
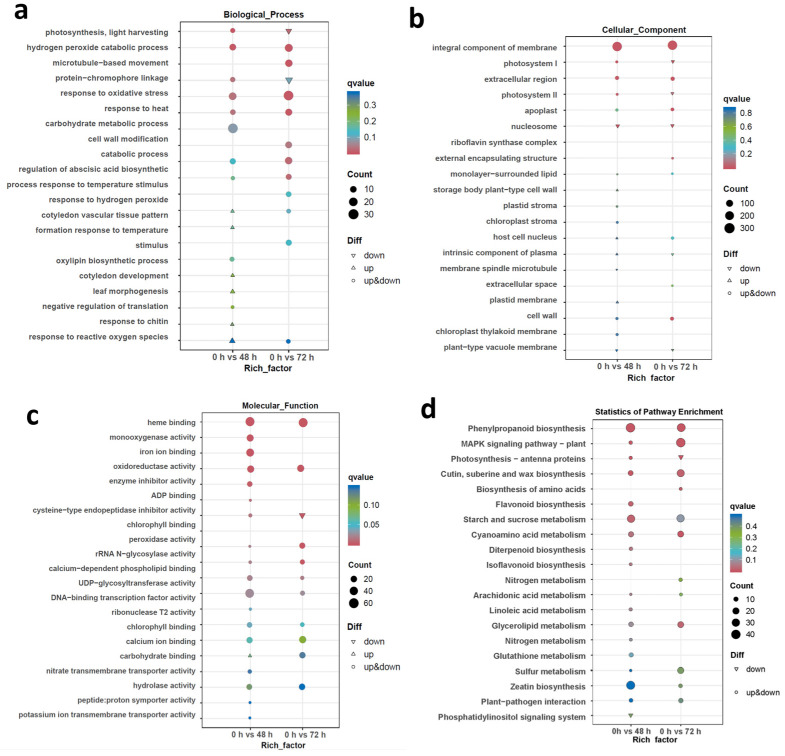
GO and KEGG enrichment analysis. (**a**–**c**) GO enrichment analysis for DEGs found in the Chaofeng F1 root after 48 h and 72 h LRT-treated vs. at 0 h: (**a**) biological process, (**b**) cellular component and (**c**) molecular function. (**d**) KEGG enrichment analysis for DEGs found in the Chaofeng F1 root DEGs between 48 h and 72 h LRT-treated vs. at 0 h. The size of the dot indicates the number of genes enriched into this pathway. The color of the dot indicates the significance of this pathway.

To illustrate functional differences in LRT-responsive genes that drove the transcriptional responses in the bottle gourd roots, a GO enrichment analysis of DEGs was performed to explore significant relevant biological processes, cellular components and molecular functions (Figure 4). In contrast to the control, the top five biological processes involved in root LRT stress for 48 h and 72 h were in response to photosynthesis, light harvesting, hydrogen peroxide catabolic processes, microtubule-based movement, protein–chromophore linkage and oxidative stress (Figure 4a). The top five of cellular component categories were commonly found after 48 h of LRT, 0 h and 72 h of LRT, and in the 0 h datasets, and included integral components of the membrane, photosystems I and II, the extracellular region, and the apoplast (Figure 4b). The top five enriched molecular functions involved in root LRT stress for 48 h and 72 h were heme binding, monooxygenase activity, iron ion binding, oxidoreductase activity and enzyme inhibitor activity (Figure 4c).

The KEGG enrichment analysis of the DEGs to elucidate the different biological processes in the bottle gourd root response to LRT stress showed that DEGs were significantly enriched in phenylpropanoid biosynthesis, mitogen-activated protein kinase (MAPK) signaling, photosynthesis, suberin and wax biosynthesis, amino acid biosynthesis, and so on (Figure 4d). Taken together, during the process of LRT stress, the bottle gourd roots evolved a series of DEGs and unique genes and pathways.

### 2.5. TF Families Respond to LRT Differentially

**Figure 5:**
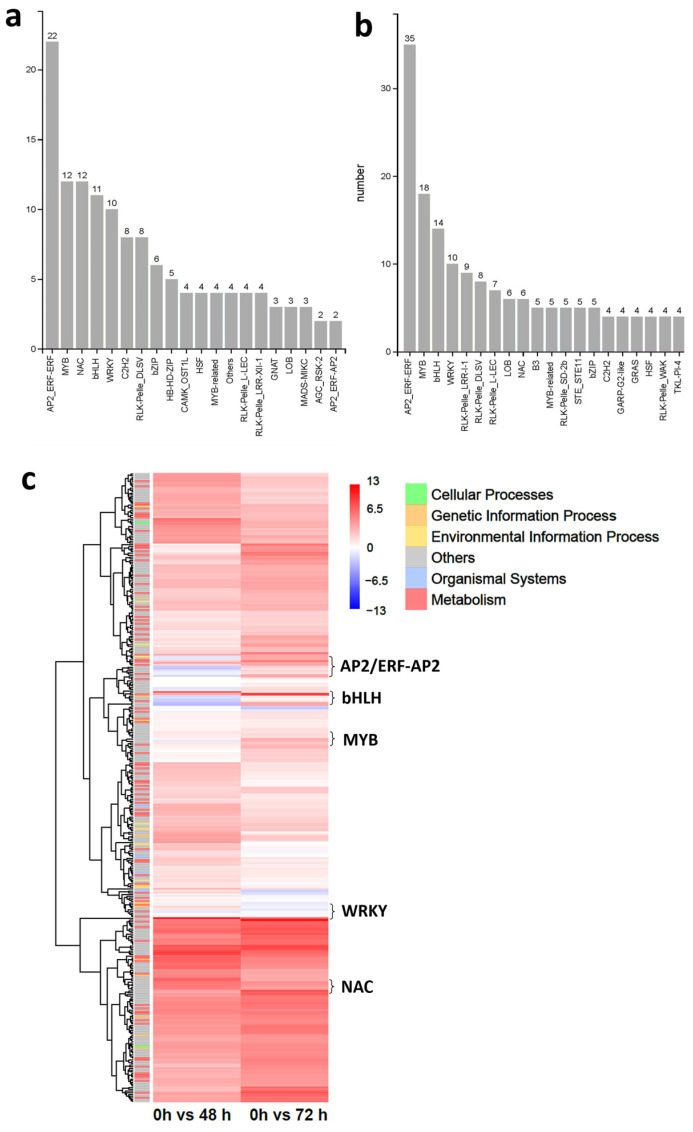
DEGs in each TF family detected in the Chaofeng F1 root after (**a**) 48 h or (**b**) 27 h of LRT vs. 0 h. (**c**) Heatmap of interspecific DEGs in part TF family expression levels and functional annotations. A fold change cutoff ≥ 2 and FDR < 0.05 were used to identify differentially expressed genes.

TFs serve as crucial regulators of LRT stress and low-temperature conditions, making them invaluable genetic resources for breeding melon crops with enhanced tolerance to low temperatures. In order to screen the DEGs of the TF-mediated LRT stress response, we analyzed the DEGs after 48 h of LRT vs. 0 h and 72 h of LRT vs. 0 h datasets; 174 TFs and 271 TFs were detected, respectively (Table S1a,b). As shown in Figure 5a, 131 TFs were differentially expressed in the comparison of 48 h of the LRT vs. the 0 h control. The expressions of 162 TFs were different under 48 h of LRT vs. the 0 h control (Figure 5b). Several TFs responded to only one treatment time point; like the HB-HD-ZIP, CAMK, GNAT and MADS were characterized as DEGs only in samples undergoing LRT for 48 h compared with the 0 h control, while B3, SET11 and GRAS were detected only in root undergoing LRT treatment for 72 h (Figure 5a,b). There were five TF families containing more than 10 DEGs found in samples treated with LRT for 48 h: AP2/ERF (22), MYB (12), NAC (12), bHLH (11), WRKY (10) (Figure 5a and Table S1a). In samples treated with LRT for 72 h, AP2/ERF (35), MYB (18), bHLH (14) and WRKY (10) were the four TF families containing more than 10 DEGs, in contrast to the control (Figure 5b and Table S1b).

**Figure 6:**
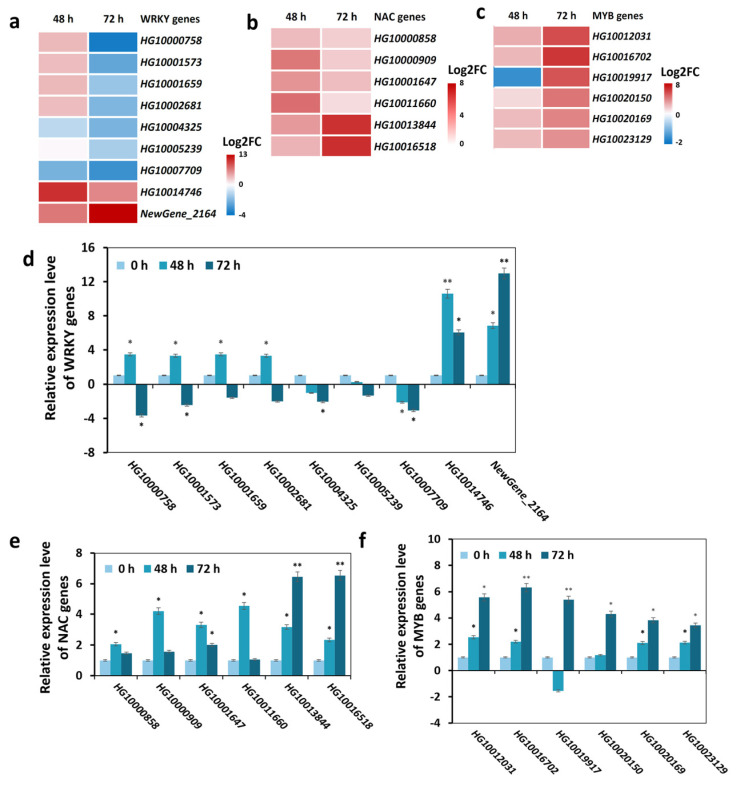
Common DEGs classified as TFs between 48 h and 72 h of LRT treatment vs. 0 h as the control. (**a**–**c**) Heatmaps showing transcript abundances of differentially expressed (**a**) WRKY, (**b**) NAC and (**c**) MYB TFs in 48 h and 72 h LRT-treated bottle gourd roots compared with those at 0 h. The log2 fold change (FC) scale is indicated next to the heatmap. (**d**–**f**) Expression analysis of (**d**) WRKY, (**e**) NAC and (**f**) MYB TFs in Chaofeng F1 roots after 48 h and 72 h of LRT treatment. In (**d**–**f**), a reference gene was used, *UBQ5* (*HG10015223*), and relative expression values were calculated using the 2^−ΔΔCt^ method. The asterisks indicate a significant difference between means (* *p* < 0.05, ** *p* < 0.01) according to Duncan’s multiple range tests.

Previous studies have demonstrated that TFs belonging to the WRKY, NAC and AP2/ERF superfamilies, among others, play pivotal roles in regulating plant root growth by regulating the levels of ROS and plant hormones in response to LRT stress [41,42,43]. A total number of 293 TFs were differentially expressed, explaining the LRT-treated phenotype of bottle gourd roots. of which we predicted the TFs and annotated the functions of DEGs, with these TF families and their expressions shown in heatmaps (Figure 5c). Here, we selected common DEGs of the AP2/ERF, bHLH, MYB and WRKY families that had significant differences in their expression trends after 48 h and 72 h of LRT compared to the control. However, the DEGs’ expression levels in the NAC family between 48 h and 72 h were significantly upregulated. Nine WRKY DEGs induced by LRT treatment showed three downregulated genes and two upregulated genes at both time points and significant differences in four genes (Figure 5a). Out of the six upregulated NAC DEGs, which were induced by LRT treatment for 48 h and 72 h compared with the 0 h control (Figure 6b), five upregulated MYB DEGs appeared at both time points, and one MYB DEG was upregulated at 48 h and downregulated at 72 h of LRT treatment (Figure S1b). The number of upregulated and downregulated DEGs in the AP2/ERF and bHLH families was not as distinct as that for the other superfamilies (Figure S1a,b). The above results suggest that bottle gourd root specimen TFs of different superfamilies respond to LRT stress in different ways, demonstrating upregulation or downregulation, earlier or later responses, and more or fewer changes at the transcriptional level, providing functional insights into their cold stress response.

### 2.6. qRT-PCR Analysis Validates the Expression Patterns of DEGs

To verify the RNA-Seq results, we selected DEGs annotated as several TFs in root specimens subjected to LRT treatment for 48 h and 72 h compared with the 0 h control for the qRT-PCR assay (Figure 6d–f). WRKY transcription factors are widely involved in plant abiotic stress [41]. As shown in Figure 6a, *HG10014746* and *NewGene_2164* were the WRKY genes whose expressions exhibited the highest induction with LRT treatment, and of these, the expression of *HG10014746* was higher after 48 h LRT treatment compared to 72 h, and the expression of *NewGene_2164* was the opposite. The qRT-PCR analysis showed that the expression patterns were the same as the RNA-Seq results (Figure 6a,d). Interestingly, *HG10014746*, also known as *LsWRKY55* and *AtWRKY55*, has the function of positively regulating leaf senescence and defense response [44], while *SbWRKY55* responds to salt stress and negatively regulates its salt tolerance [45]. Furthermore, *NewGene_2164* was highly homologous to *AtWRKY75*, and *AtWRKY75* controls salt stress tolerance in *Arabidopsis* [46]. Hence, our study discovered new functions of *HG10014746* and *NewGene_2164* in response to LRT stress, in addition to its other abiotic stress.

Moreover, NAC and MYB are essential TF families in plant response to abiotic stress. qRT-PCR confirmed that LRT treatment positively regulated the expression of six NAC superfamily DEGs (*HG10000858*, *HG10000909*, *HG10001647*, *HG10011660*, *HG10013844* and *HG10016518*) and six MYB superfamily DEGs (*HG10012031*, *HG10016702*, *HG10019917*, *HG10020150*, *HG10020169* and *HG10023129*) (Figure 6e,f). Notably, the expression of *HG10013844* (*NAC1*), *HG10016518* (*NAC45*), *HG10012031* (*MYB15*) and *HG10016702*(*MYB70*) were more significantly upregulated under LRT stress than the control. Additionally, all four genes exhibited higher expression levels after 72 h of LRT treatment compared to those at 48 h. Previous studies have highlighted the functional roles of these genes; for example, *AtNAC1* regulates root ground tissue maturation in *Arabidopsis* [36], *OaNAC45* alleviates cold-induced root growth inhibition [47], *SlMYB15* positively regulates cold tolerance through the CBF pathway [48] and *AtMYB70* modulates root system development in *Arabidopsis* [49]. These findings suggest that these DEGs play vital roles in root growth and low-temperature stress responses.

Furthermore, we selected several AP2/ERF and bHLH genes to validate the RNA-Seq data through qRT-PCR assays; homologous genes of those in other crops have not been functionally characterized. We observed a high consistency in RNA-Seq and qRT-PCR data; for example, two AP2/ERF genes (*HG10009058* and *HG10012026*) and two bHLH genes (*HG10006451* and *HG10007116*) showed increased expression levels after 48 h and 72 h of LRT treatment compared with the 0 h control (Figure S1a,b). However, eleven AP2/ERF genes (*HG10004041*, *HG10007892*, *HG10009058*, *HG10012026*, *HG10012533*, *HG10013383*, *HG10015512*, *HG10016523*, *HG10017319*, *HG10018601* and *HG10019304*) and four bHLH genes (*HG10006168*, *HG10010192*, *HG10010412* and *HG10010574*) were less induced after 48 h LRT treatment than in the 0 h control, but had a higher expression level after 72 h LRT treatment (Figure S1c,d).

Taken together, through the qRT-PCR analysis of 39 selected DEGs, their expression patterns were confirmed in response to LRT treatment, aligning closely with the RNA-Seq data. These credible candidate genes, responsive to LRT stress, will not only extend our comprehension of the functions of known TFs but uncover novel TFs in bottle gourd root specimens that are involved in LRT responses.

## 3. Discussion

Low-temperature tolerance causes multiple effects in root growth and crop development, representing a complex trait controlled by multiple genes and largely influenced by environmental conditions [9,50]. Plant TFs occupy a pivotal position in regulating plant growth and development and responding to low-temperature stress. These TFs modulate RSA by regulating multiple genes or plant hormones involved in the low-temperature response pathway, thereby endowing plants with the capacity to tolerate lower temperatures. Proper genetic modification of TFs holds potential for breeding cold-tolerant bottle gourd type rootstock. However, due to the relatively recent commencement of molecular biology research in the bottle gourd, there remains a dearth of studies on the response of TFs to low-temperature stress and the regulation of root traits in this species, and there is a lack of correlational analysis and regulation mechanisms. Therefore, we screened and identified 39 DEGs from five TF families by combining RNA-Seq analysis with qRT-PCR assays targeting the bottle gourd root specimen under 15 °C LRT stress.

### 3.1. LRT Stress Affected the Phenotype and Physiology of Bottle Gourd Roots

Plant root systems are vital organs responsible for efficient water and nutrient uptake as well as stress perception in both the atmosphere and soil [4]. Low-temperature stress alters root system architecture, subsequently impacting various root functions ranging from the acquisition of nutrients to hydraulic conductance [51,52]. In our study, the effects of long-term LRT stress on bottle gourd root trait changes were evident both phenotypically and physiologically (Figure 1 and Figure 2). Specifically, we observed that the lateral root length was significantly reduced after exposure to 15 °C LRT stress (Figure 1a). Concurrently, root activity decreased significantly with the increase in LRT stress (Figure 1b,c). The MDA content and relative electrolyte permeability are key indicators for assessing plasma membrane peroxidation and changes in cell membrane permeability under stressful conditions. Notably, the MDA content and relative electrolyte permeability in the bottle gourd root specimens significantly increased after 48 h of LRT stress (Figure 2a,b). These results demonstrated the similar physiological and phenotypical responses of bottle gourd roots to LRT treatment, which aligns with observations in other crop species exposed to low-temperature stress [53,54,55].

### 3.2. Dynamic Changes in ROS Levels Occurred under LRT Conditions

Root development is also affected by ROS, which are an essential component of plants in response to various stresses [34,56]. They are increasingly being recognized for their crucial physiological functions in plant growth and stress responses. Moderate levels of ROS can regulate plant responses to various stresses by participating in signal transduction pathways, while excessive ROS can lead to plants entering an oxidative stress state [9]. In addition, the regulation of root elongation has been attributed to ROS gradients [3]. The production of ROS during cold stress can lead to damage in roots, as illustrated by higher levels of H_2_O_2_ and O^2−^ accumulation in cold-sensitive plant genotypes [57]. To investigate the effect of LRT inhibition on oxidative damage to the plant photosystem in detail, we detected a significant increase from 36 h in the H_2_O_2_ and O^2−^ content in the bottle gourd root after 15 °C LRT compared with the control (Figure 2c,d). These results aligned fully with the previous reports on the ROS content in pepper and tomato roots after LRT stress, in which we demonstrated that the induction of ROS levels in the bottle gourd root increased significantly after 36 h of LRT [6,58].

SODs, PODs and NADPH oxidases are the main ROS produced in plants [3,59]. SOD and POD activities were significantly enhanced after 48 h under LRT stress, and the highest activities of both enzymes were demonstrated after 72 h of LRT treatment in contrast to the 0 h control (Figure 2e,f), in agreement with reports on the sweet potato and tomato [6,60]. In addition, the SOD and POD activities had the same increasing trends under LRT stress. Our results showed that LRT initially increased the H_2_O_2_ and O^2−^ contents to cause severe damage to the bottle gourd roots’ development and then also increased the SOD and POD activities, eliminating the excess accumulation of ROS to protect against oxidative damage and eventually improving the tolerance to LRT stress.

### 3.3. Differentially Expressed TFs of the Bottle Gourd Root in Response to LRT Stress

Plant TFs mediate cold responses via the direct or indirect regulation of hormone signaling, ROS, metabolites, and other cold-responsive pathways [42,44]. TFs (such as WRKY, NAC and MYB, etc.) regulate root architecture by regulating multiple genes or plant hormone levels in a low-temperature response pathway, thereby giving plants the ability to tolerate low temperatures [61]. A total of 174 TFs were differentially expressed in the root samples treated with LRT for 48 h compared with the 0 h control (Figure 5a). By contrast, we identified 271 DEGs annotated as TFs in the bottle gourd root after 72 h of LRT treatment (Figure 5b). Among them, AP2/ERF, MYB, NAC, bHLH, C2H2 and WRKY were identified in both test groups, and they have previously been reported in the study of low-temperature stress and/or regulated root traits [62]. Whether or not TFs of the B3, HB-HD-ZIP, CAMK and other families play roles in the response to LRT stress or the regulation of root traits has not been reported. Furthermore, we performed qRT-PCR assays to examine the expression levels of 39 TFs identified by RNA-Seq validation, and the results were consistent with the accuracy of the transcriptomic sequencing results. Overall, we suggest that these TFs could have responded to LRT stress and adjusted their expression-controlled root phenotypes to resist low-temperature stress. Our study thus provides illuminating insights into the regulatory networks of the LRT response in bottle gourd roots.

MYB-type TFs are important regulators of plant tolerance to cold stress involved in regulating *CBF* expression. MYB15 allows for the precise regulation of the expression and subsequent cold tolerance of CBF genes, which are rapidly and highly induced by responding to low temperatures [47]. We also identified MYB15 in the RNA-Seq of the bottle gourd root. *LsMYB15* (*HG10012031*) responded to LRT and its expression was upregulated after 48 h and 72 h LRT treatment (Figure 6c,f), which were previously reported to positively respond to low-temperature stress in *Arabidopsis* and the tomato [63]. Moreover, other significantly differentially expressed MYB TFs genes (*HG10016702* and *HG10019917*) were also identified by RNA-Seq and qRT-PCR analyses.

WRKY TFs simultaneously regulate stress resistance and plant-specific growth and development [64]. WRKYs also participate in plant maturation by root elongation, lateral root formation and root hair patterning [41]. Furthermore, WRKY has diverse regulatory effects on crops, such as, SlWRKY33 enhances cultivated tomato cold resistance by directly targeting and inducing transcription factors and molecular chaperone genes, but GhWRKY22 (an ortholog of *Arabidopsis thaliana* AtWRKY33) participates in regulating anther/pollen development in cotton [65,66]. As shown in Figure 6a,b, we examined the expression levels of ten differentially expressed WRKYs identified by RNA-Seq and confirmed two DEGs (*LsWRKY55*, *HG10014746* and *NewGene_2164*) that were significantly upregulated due to LRT treatment by 6–12-fold. *NewGene_2164* was highly orthologous in *AtWRKY75*. As previous studies have indicated, PdeWRKY75 regulates the development of adventitious roots by modulating hydrogen peroxide content in the poplar, AtWRKY75 regulation of root hair-patterning genes and controlling salt stress tolerance [50,67,68]. *SbWRKY55* responds to salt stress and negatively regulates its salt tolerance [49]. As shown above, we obtained differentially expressed TFs under LRT stress. Among them, we indicated many DEGs of some TFs under LRT stress that regulated root traits that had not yet been reported. Thus, our study has illuminating insights into the regulatory networks of LRT response in the bottle gourd root.

In addition to the above results, we obtained differentially expressed TFs under LRT stress. Notably, the involvement of differentially expressed TFs under LRT stress in regulating root traits has not yet been reported. Therefore, our study can provide reference and inspiration for a deeper understanding of the regulatory network of bottle gourd roots in response to LRT stress and the control of root traits. This will provide references for future research to discover new target genes for improving the low temperature and LRT tolerance of bottle gourd-type rootstock cultivars.

## 4. Materials and Methods

### 4.1. Plant Materials, 15 °C LRT Treatment and Sample Preparation

The bottle gourd variety Chaofeng F1 (low-temperature-tolerant) was used in this study. Bottle gourd seeds were placed in vermiculite with 75% water content and germinated at 30 °C for 2–3 days. When the roots had grown to 2–3 cm, they were cultured in sterilized seed germination pouches (Cat. No.: CYG-38LB; Phytotc, Beijing, China, http://www.phytotc.com/). The pouches were kept in a controlled growth chamber for a 16 h/26 °C day and 8 h/18 °C night cycle, at 250 μmol m^−2^ s^−1^ and at a relative humidity of 70%. After 5 days, they were placed in a low-constant temperature water bath (DC-0506; Shunma Tech, Linyi, China) for 15 °C LRT treatment. At each time point, the root phenotype was recorded using a scanner (WinRHIZO; Zhejiang, China), and the PR and LR samples were taken for the determination of physiological and biochemical indexes. The root samples collected at 0 h, 24 h, 36 h, 48 h and 72 h were used for determining physiological and biochemical indexes. Furthermore, root samples at 0 h, 48 h and 72 h were frozen in liquid nitrogen and stored at −80 °C for subsequent RNA-Seq and qRT-PCR experiments. Three biological replicates were included for each time point of each treatment.

### 4.2. Cell Viability Staining

Plants were treated with 75 mM NaCl for 48 h, and then the cell viability of the roots (PR and LR apex zones) was assessed using the fluorescein diacetate (FDA)–propidium iodide (PI) double-staining method as described by Chakraborty (2016) [69]. The FDA dye solution (final concentration: 100 μg/mL) and PI dye solution (final concentration: 10 μg/mL) were mixed; that is, 10 μg of FDA and 10 μg of PI were added to 10 mL of 0.65 mol/L mannitol. They were dyed for 15–30 min at room temperature in the dark followed by three washes. A laser confocal scanning microscope (TauSTED Xtend; Leica, Wetzlar, Germany) was used to observe and take pictures. Fluorescence intensity images were quantified using ImageJ software, version 7.12.

### 4.3. Determination of Relative Electrolyte Leakage

The relative electrolyte leakage determination method was carried out with reference to Jambunathan (2010) [70]. The plant roots were placed in a centrifuge tube containing 20 mL of distilled water, and the conductivity of the distilled water was measured and recorded as EC_0_. Samples were shaken on a shaker (200 r/min) for 2 h at room temperature and subsequently placed for 10 in a water bath at 100 °C min, followed by adjustment to room temperature, and the conductivity of the root solution was measured at this time, which was recorded as EC_1_. The water bath was set at 100 °C for 10 min, and the samples were then cooled to room temperature. Samples were repeatedly shaken for 2 h on the shaker and the conductivity of the root solution was measured and recorded as EC2: REP = (EC_1_ − EC_0_)/(EC_2_ − EC_0_) × 100%.

### 4.4. Determination of Malondialdehyde (MDA) and Peroxide Enzyme Activity

The determination of MDA content was performed using an MDA Content Assay Kit (Solarbio, Beijing, China). For the quantification of H_2_O_2_ and O^2−^ content, an assay kit (JC DETECT, Nanjing, China) was used. POD and SOD activities were determined using a Peroxidase (POD) Activity Assay Kit and Superoxide Dismutase (SOD) Activity Assay Kit (WST-1 method; Solarbio, Beijing, China). According to the manuals of the MDA, H_2_O_2_, O^2−^, SOD and POD assay kits, the absorbance of each sample at different wavelengths was determined using a Cary 5000 ultraviolet–visible–near-infrared (UV–Vis–NIR) spectrophotometer (Agilent, Santa Clara, CA, USA), and calculation was carried out.

### 4.5. Total RNA Extraction

Total RNA was extracted using a Fast Pure Cell/Tissue Total RNA Isolation Kit V2 (Vazyme, Nanjing, China). RNA degradation and contamination were monitored on 1.5% agarose gels. RNA purity and concentration were measured using a NanoPhotometer^®^ spectrophotometer (IMPLEN, Westlake Village, CA, USA). RNA integrity was assessed using a NanoDrop™ 8000 Assay Kit (Agilent Technologies, Santa Clara, CA, USA).

### 4.6. Library Construction and RNA-Seq Data Analysis

RNA library preparation and sequencing were performed at Beijing Biomarker Technologies Co., LTD.; Beijing, China (http://www.biomarker.com.cn/). RNA-Seq analysis was conducted with three biological replicates per sample using the Illumina NovaSeq6000 sequencing platform (Illumina, San Diego, CA, USA). After the sequencing data were downloaded, the bioinformatics analysis process provided by BMKCloud; Beijing, China (https://www.biocloud.net/) was used for data analysis. The reference genome version was *Lagenaria siceraria* cv. Hangzhou Gourd. Paired-end clean reads were aligned to the reference genome using HISAT2, Version HISAT2 2.2.1, and StringTie, Version 2.2.0 was used to assemble and splice reads. The alternative splicing types and corresponding expression levels of each sample were obtained using by ASprofile, Version 1.0.4. DIAMOND combined with the Non-Redundant (NR), Swiss-Prot, Clusters of Orthologous Groups (COG), FuKaryotic Orthologous Groups (KOG), and the KEGG database alignment results were used to annotate new genes. DESeq2 was used for differential analysis. During the detection of DEGs, a fold change ≥ 2 and FDR < 0.05 were used as screening criteria. Common differentially expressed TFs in bottle gourd root after 48 h and 72h LRT compared with 0 h control, as determined by RNA-seq are shown in Table S2.

### 4.7. Quantitative Real-Time PCR (qRT-PCR) for RNA-Seq Validation

qRT-PCR assay was performed according to the method described by Liu (2020) [70]. The primer sequences are shown in (Table S3). The qRT-PCR was performed with a GoTaq^®^ PCR Master Mix (Promega, Madison, WI, USA) on an ABI 7500 real-time PCR system (Applied Biosystems, Foster City, CA, USA). The qRT-PCRs were performed with three biological replications and three technical replicates, and relative expression values were calculated using the 2^−ΔΔCt^ method, using the reference gene, *UBQ5* (*HG10015223*).

### 4.8. Statistical Analysis

All samples were analyzed in triplicate, and the data are expressed as the mean ± SD, unless noted otherwise. Statistical significance was determined at the 0.05 (*) and 0.01 (**) levels. All experiments were repeated at least twice with three biological replicates each time.

## 5. Conclusions

In this study, we investigated the phenotypical and physiological alterations of bottle gourd roots following LRT treatment at various intervals: 0 h, 12 h, 24 h, 36 h, 48 h and 72 h. The results demonstrated that the most profound damage occurred after 48 h of LRT stress, which led to a significant increase in electrolyte leakage, MDA content and peroxide enzyme activity, while the activities of the root cell were noticeably reduced. We subsequently validated TF families that were identified by RNA-Seq data including WRKY, NAC, MYB, bHLH and AP2/ERF, using qRT-PCR. Overall, this study identified novel LRT-responsive TFs that have the potential to serve as target genes for breeding bottle gourd rootstock varieties with improved tolerance to low-temperature stress.

## Data Availability

Data are contained within this article and the Supplementary Materials.

## Supplementary Materials

The following supporting information can be downloaded at: https://www.mdpi.com/article/10.3390/ijms25158288/s1.
