# Supplementary material for: A Transcriptomic Analysis of Bottle Gourd-Type Rootstock Roots Identifies Novel Transcription Factors Responsive to Low Root Zone Temperature Stress
Source: Int J Mol Sci. 2024 Jul 29;25(15):8288. doi: 10.3390/ijms25158288 (PMC11313094; doi:10.3390/ijms25158288)
Supplement: Supplementary file 1 [file ijms-25-08288-s001.zip › ijms-3100621-supplementary figure.pdf]

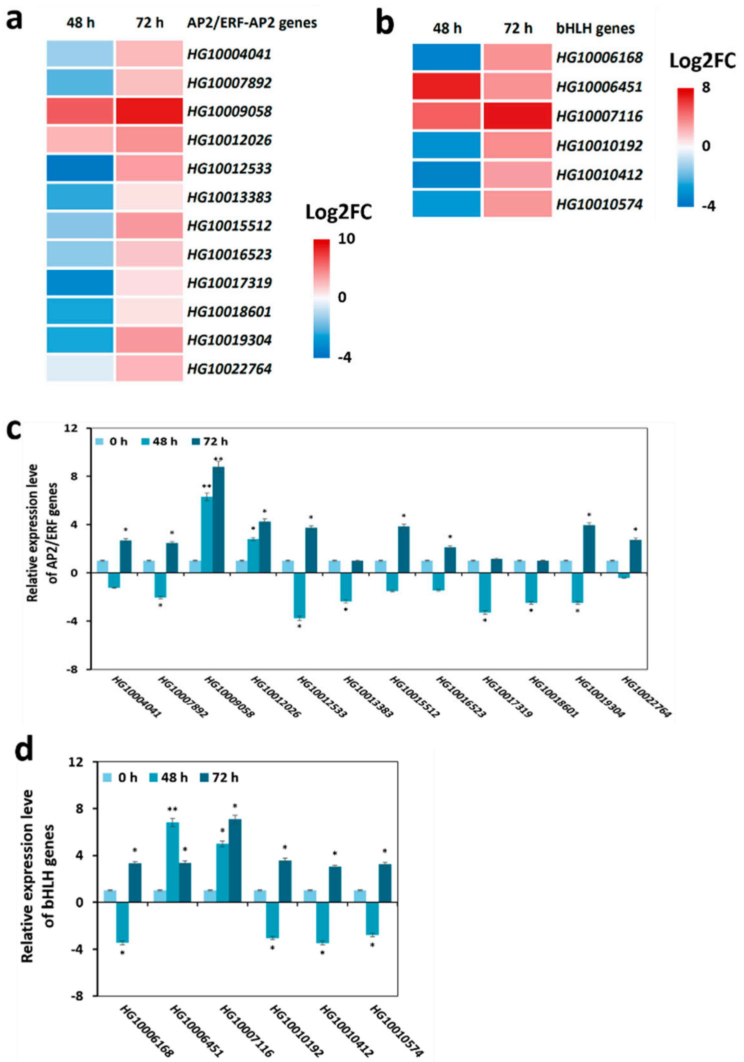

Figure S1. Common DEGs classified as TFs between 48 h and 72 h LRT treatment vs. 0 h control. (**a**,**b**) Heatmaps show transcripts abundances of differentially expressed AP2/ERF (**a**), bHLH (**b**) in 2 h and 4 h dehydration-treated MT leaves compared with 0 h. The log2 fold change (FC) scale is indicated next to the heatmap. (**c**) Expression analysis of AP2/ERF, (**d**), bHLH TFs in Chaofeng F1 roots after 48 h, and 72 h LRT treatment. (**c**,**d**), reference gene: *UBQ5* (*HG10015223*), relative expression values were calculated using the $2^{-\Delta\Delta Ct}$ method. Letters indicate a significant difference between means (P<0.05) according to Duncan's multiple range test.
